# Supplementary material for: Indicators, Goals, and Assessment of the Water Sustainability in China: A Provincial and City—Level Study
Source: Int J Environ Res Public Health. 2023 Jan 30;20(3):2431. doi: 10.3390/ijerph20032431 (PMC9915312; doi:10.3390/ijerph20032431)
Supplement: Supplementary file 1 [file ijerph-20-02431-s001.zip › ijerph-2166609-supplementary.pdf]

International Journal of
Environmental Research and Public Health

MDPI

Supplementary material S1. Research framework.

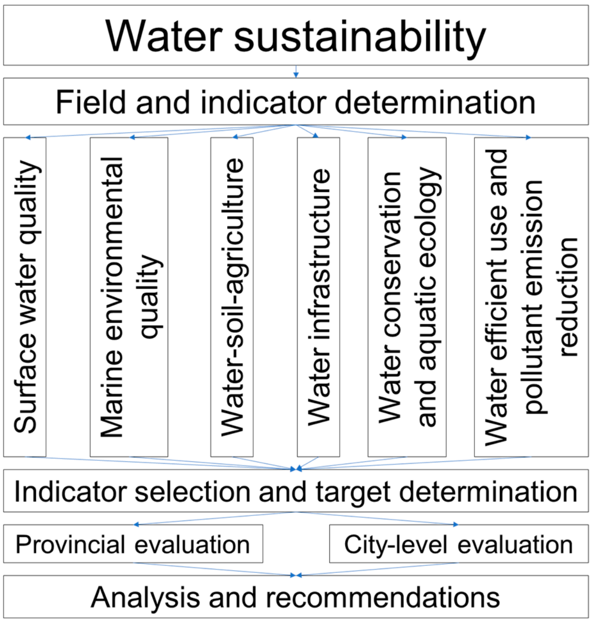

Figure S1. Research framework.

Supplementary material S2. Indicator interpretation and basis for target determination.

Indicator interpretation:

For indicator 1–3, 5–7, and 10, the grades is evaluated according to China's Environmental quality standards for surface water (GB 3838-2002), Standard for groundwater quality (GB/T 14848-2017), Water quality standard for drinking water sources (CJ 3020-93), and Sea water quality standard (GB 3097-1997). Indicator 4 is according to China's Water Function Zoning of Important Rivers and Lakes in the Country (2011-2030). For indicator 11, effective utilization coefficient of irrigation water refers to the ratio of the effective amount of water irrigated into the field to the amount of water introduced by the head of the canal. For indicator 21, the nature protected area refers to land or sea areas delineated or confirmed by governments to implement long-term protection of important natural ecosystems, natural relics, natural landscapes and the natural resources, ecological functions and cultural values. For indicator 22 and 24, the ecological protection red-line refers to the spatial boundaries and management limits that need to be strictly protected in terms of natural ecological service functions, environmental quality and safety, and natural resource utilization, so as to maintain national and regional ecological security and sustainable economic and social development, and protect the health of the people. For indicator 25, the ecological environment index refers to the synthesis of a series of indexes that reflect the ecological environment quality of the assessed area, incouding biological abundance index, vegetation cover index, water network density index, land stress index, pollution load index, and environmental restriction index.

Indicator goal determination:

We determined the goals for indicators 1–7, 10, and 27 mainly referred to the national plans for water ecological environment protection and the water safety of China. The 2035 goals for indicators 8 and 9 were according to China's ecological civilization standards for villages and towns. The 2035 goals for indicators 11–12 and 31–32 referred to the

description of SDGs and the good performance worldwide and nationwide. For the water infrastructure indicators, the 2035 goals mainly referred to the national plans for ecology and environment protection, urban and rural infrastructure construction, and zero-waste city. We set the 2035 goals for indicators 19 to 24 according to China's construction plan for major projects of ecological protection and restoration support systems while referring to the other documents, such as ecological protection red-line delineation. We mainly considered the good performance nationwide when setting the 2035 goal for indicator 25, and we also referred to the methods and country-specific results of the Environmental Performance Index. The 2035 goal for indicator 26 was according to China's plans for natural ecological protection and soil and water conservation. The 2035 goals for indicator 28-30 referred to China's ecology and environment protection plan.

Table S1. Sources and references of the goals.

Goals	Source and reference
1–7, 10, and 27	1. Water Pollution Prevention and Control Action Plan<br>2. The 14th Five-Year Plan for the National Economic and Social Development and Outline of Long-term Goals for 2035 of China<br>3. Assessment Method for Implementing the Most Stringent Water Resources Management System<br>4. Comprehensive National Water Resources Plan<br>5. National Water Conservation Action Plan<br>6. The 14th Five-Year Plan for Soil, Groundwater and Rural Ecological Environment Protection<br>7. The 14th Five-Year Plan for Ecological and Environmental Protection
8 and 9	National Ecological Civilization Construction Demonstration Villages and Towns Indicators (Trial)
11–12 and 31–32	1. National Water Conservation Action Plan<br>2. Xu et al., 2020<br>3. Wang et al., 2022<br>4. National Bureau of Statistics
13–18	1. National Ecological Civilization Construction Demonstration Cities and Counties Construction Index<br>2. National Ecological Civilization Construction Demonstration Villages and Towns Indicators (Trial)<br>3. Water Pollution Prevention and Control Action Plan
19–24	1. Construction Plan for Major Projects of Ecological Protection and Restoration Support Systems<br>2. Plan for Ecological Protection Red-line Delineation
25	1. Wendling et al., 2022<br>2. National Ecological Civilization Construction Demonstration Cities and Counties Construction Index
26	1. The 14th Five-Year Plan for Ecological and Environmental Protection<br>2. National Soil and Water Conservation Plan
28–30	The 14th Five-Year Plan for Ecological and Environmental Protection

Reference

1. Xu, Z., Chau, S.N., Chen, X., Zhang, J., Li, Y., Dietz, T., Wang, J., Winkler, J.A., Fan, F., Huang, B., Li, S., Wu, S., Herzberger, A., Tang, T., Hong, D., Li, Y., Liu, J. Assessing progress towards sustainable development over space and time. *Nature* **2020**, *577*, 74–78. https://doi.org/10.1038/s41586-019-1846-3.
2. Wang, Q., Liu, C., Hou, Y., Xin, F., Mao, Z., Xue, X. Study of the spatio-temporal variation of environmental sustainability at national and provincial levels in China. *Sci. Total. Environ.* **2022,** *807*, p.150830. https://doi.org/10.1016/j.scitotenv.2021.150830.
